# Supplementary material for: Costs of providing HIV care and optimal allocation of HIV resources in Guyana
Source: PLoS One. 2020 Oct 29;15(10):e0238499. doi: 10.1371/journal.pone.0238499 (PMC7595312; doi:10.1371/journal.pone.0238499)
Supplement: Attachment — Submitted filename: Response to Reviewers 1.docx [file pone.0238499.s001.docx]

# Response to reviewer #1

We thank the reviewer for the thoughtful comments provided. Our responses are given below.

| **Comment** | **Response** |
| --- | --- |
| While variance in cost between sites may be an opportunity for efficiency, I wondered if different clinics served different populations, had different viral load suppression rates and as such had different human resource needs. | This is an excellent point, and we have added a point on this to the first paragraph of the discussion section. |
| Consideration could be given to including the limitations of this analysis. Eg are we assuming that loss to follow up in this analysis is included or excluded. | We have reoriented the discussion section so that the study limitations are clearer, and in particular clarified this question about LTFU. |

# Response to reviewer #2

We thank the reviewer for the thoughtful comments provided. Our responses are given below.

| **Comment** | **Response** |
| --- | --- |
| This is an interesting paper that reports in great detail the results of a microcosting exercise to calculate unit costs along the HIV care cascade in Guyana. The unit costs are then used to parametrise a resource allocation model looking at two possible scenarios (historical budget allocation vs reallocation to optimise incidence and mortality outcomes). While the paper’s reporting on the costing exercise methods, results and possible limitations is thorough and comprehensive, there is comparatively quite limited space devoted to explaining the methods and assumptions underlying the resource allocation modelling. | We thank the reviewer for the summary and positive words. We have devoted more attention to explaining the methods and assumptions on the resource allocation modelling; more details are provided in the responses to the comments below. |
| There are two main concerns in this respect. Firstly, it is not clear why the authors chose to explore those two particular scenarios in the model. Optimal vs historical allocation seem to be the best and worst case, respectively, which is definitely interesting for exploring any resource gaps. However, there is no indication as to whether any of these scenarios is actually realistic or perhaps other more realistic scenarios could have been explored. Did the authors consider expert elicitation or validate these scenarios with local stakeholders in the budgeting process? And, if not, how were the scenarios chosen and why? | This is a fair point. The scenarios were indeed chosen in consultation with local stakeholders, several of whom are authors on the paper or listed in the acknowledgements. We have added an extra sentence on this to the end of the methods section. |
| Perhaps the overall aim of the analysis should be clarified, particularly in terms of the perspective and intended audience for the allocative efficiency analysis. In the discussion, the it is stated that the analysis shows that there are vulnerabilities in the HIV financing model in Guyana due to reliance on donor funding (lines 263-264). This is not emerging clearly in the analysis and perhaps could have been further explored in the model? | We agree that the statement about having found vulnerabilities in the sustainability of the response was not a conclusion of our work. We have removed that sentence and reframed it as a more general discussion point. |
| Secondly, there is no mention throughout the manuscript of how uncertainty was treated in the model and no intervals are reported for the epidemiological parameters and results. Although the analysis uses a published, referenced model, this information is vital for assessing the reliability of model results. | We have devoted a significant part of the paragraph on limitations of this study to a discussion of why uncertainty estimates were not included in this work. We regret that they could not be included, but we feel that would go beyond the abilities of the data to attempt to quantify the uncertainty here. |
| Line 41: the term PLHIV was never defined | We have added a definition |
| Lines 64-65: were the facilities randomly selected? | The facilities were purposively selected. We have provided additional detail. |
| Line 91: capital costs is a more standard term than “investment costs” and it is used below. I suggest changing to capital costs in every instance for clarity and consistency | We have changed this as suggested |
| Line 123: “geographical optimization algorithm”, fine to refer to source publication but perhaps helpful for readers to include here a brief explanation, at least of the criteria used to optimize. | We have added this. |
| Line 170: “weighted average”, consider mentioning the weighting formula in the methods. | We have provided the explanation on the calculation of weighted average. |
| Lines 218-221: this sentence is quite long and may be missing some punctuation In general, there are several unclear sentences that appear to be missing punctuation or clauses. | We have edited this sentence and proofread the text throughout. |
| Table 5 is not easy to read and, in general, presenting median costs is not a standard requirement. I would suggest removing the median and presenting mean and standard deviation on the same line, as well as switching rows and columns in the table to make it more compact. | We have revised Table 5 as suggested. |
